# Human Blood IgA^+^ Memory B Cells Differ From IgG^+^ B Cells by Expressing Gut‐Homing and Regulatory Markers

**DOI:** 10.1002/eji.70191

**Published:** 2026-04-26

**Authors:** Louise Le Gal, Léo Boussamet, Alexandra Garcia, Jérémy Morille, Emilie Dugast, Arnaud B. Nicot, David‐Axel Laplaud, Laureline Berthelot

**Affiliations:** ^1^ Nantes Université, Inserm, CHU de Nantes Center For Research in Transplantation and Translational Immunology CR2TI Nantes Université Nantes France; ^2^ Neurology Department CHU de Nantes Nantes France

**Keywords:** IgA, IgG, memory B lymphocytes, spectral flow cytometry, transcriptomic

## Abstract

IgA is the most produced immunoglobulin by the human body, mainly in secretions, and it exerts an important role in the activation and regulation of the immune system. Despite its importance, the phenotype of circulating IgA^+^ B cells is poorly described. Here, we deeply explored the phenotype of IgA^+^ memory B cells compared with IgG^+^ memory B cells from healthy donors using spectral flow cytometry. A bulk transcriptomic analysis was performed on isolated IgA^+^, IgG^+^ and IgM^+^ memory B cells. IgA2^+^ memory B cells expressed more gut‐homing markers (CCR9, CCR10, integrin α4β7) and CD11b, GPR183, whereas IgG^+^ B cells expressed more CXCR3 and VLA4 at the protein level. Three other markers were discriminant: CD43 and PD‐L1 were more expressed by IgA^+^ B cells, and CD25 associated with IgG^+^ B cells. The transcriptomic analysis of circulating IgA^+^, IgG^+^ and IgM^+^ memory B cells showed a particular signature of IgA^+^ B cells close to IgG^+^ B cells and highly different from IgM^+^ B cells. The transcription factor *RUNX2* was upregulated in IgA+ B cells at the mRNA and protein level. Overall, we showed that IgA^+^ memory B cells in blood carry specific markers that may help to better understand IgA^+^ B cell biology.

## Graphical abstract

Compared with IgG^+^ and IgM^+^ memory B cells, IgA^+^ memory B cells displayed distinct phenotypical markers: PD‐L1, GPR183, CCR9, CCR10, α4β7, CD11b. A particular transcriptomic signature, with *RUNX2, ARHGEF10L, XYLT1, TRPS1*, and *CHL1* upregulation, characterized the IgA^+^ memory B cell population, whereas *miR4432HG* was a marker of IgG^+^ memory B cells.

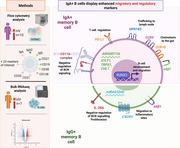

## 1 Introduction

Immunoglobulin A (IgA) is the most abundantly produced antibody class in the human body. IgA is the second Ig found in circulation after IgG and the first Ig secreted at the mucosal surface, especially in the respiratory and gastrointestinal tracts. IgA+ plasma cells can be generated in the gut‐associated lymphoid tissues (GALT), in the Peyer's patches, and in the mesenteric lymph nodes [1]. The abundance and composition of microbiota can directly influence IgA production [2]. IgA also plays a major role in immune defense at mucosal surfaces, as it can recognize a wide range of microbial antigens. They help maintain intestinal homeostasis by regulating microbiota composition, limiting expansion of several pathogens [3], and promoting immune tolerance [4, 5]. Indeed, germ‐free mice produce less IgA [3], and patients who underwent a GALT removal exhibit lower IgA secretion compared with those who did not [6]. So, IgA class switch and IgA secretion are linked to microbiota presence and stimulation in the mucosa.

There are two IgA subclasses: IgA1 and IgA2. The main differences between these two subclasses lie in the hinge region structure, which is longer for IgA1, and their distribution within the human body [7]. Serum IgA is predominantly represented by the monomeric IgA1 subclass, while in external secretions, IgA is mostly found in its dimeric form, with a higher proportion of IgA2. Serum IgA mainly originates from the production of IgA+ plasma cells in the bone marrow, whereas polymeric IgA is produced at the mucosal surface [8]. The differential interactions of monomeric and polymeric IgA contribute to their distinct distribution and functions within the human body. Moreover, binding of IgA to its Fc receptor (FcαRI) expressed by myeloid cells can lead to an anti‐inflammatory response [9, 10, 11] when IgA is monomeric or a proinflammatory response with polymeric IgA [12, 13]. Numerous studies also highlighted the IgA contribution in inflammatory and autoimmune diseases [14], such as rheumatoid arthritis [15], inflammatory bowel disease [16, 17], and IgA nephropathy [18, 19]^.^

Although IgA and IgA^+^ secreting B cells seem to exert an inflammatory role in particular autoimmune diseases, IgA^−^ producing B lymphocytes also exhibit regulatory functions, as they are known to produce anti‐inflammatory cytokines such as IL‐10 [20]. In multiple sclerosis, IgA^+^ plasmablast/plasma cells from the gut can migrate into the brain, where they regulate inflammation and secrete IL‐10 [4, 21]. Moreover, due to their multiple properties at the microbiome‐immunity interface, IgA^+^ B cells are key players in human health.

Despite these roles of IgA and IgA^+^ B cells in biological processes, the phenotype and transcriptomic profile of IgA^+^ B cells are poorly described. A more detailed characterization of this IgA^+^ B lymphocyte population is therefore necessary to better understand its role in inflammation and regulation. To address this, we have designed a multiparameter spectral flow cytometry analysis of circulating IgA^+^ B cells using 34 different markers. We also sorted IgA^+^ memory B cells and analyzed their transcriptomic profile by bulk RNA sequencing. IgA^+^ memory B cells expressed different homing and regulatory markers compared with IgG^+^ memory B cells. The transcription factor RUNX2 was exclusively expressed by IgA^+^ B cells.

## 2 Results

### 2.1 Unsupervised Analysis of Memory B Cell Marker Expression

**FIGURE 1:**
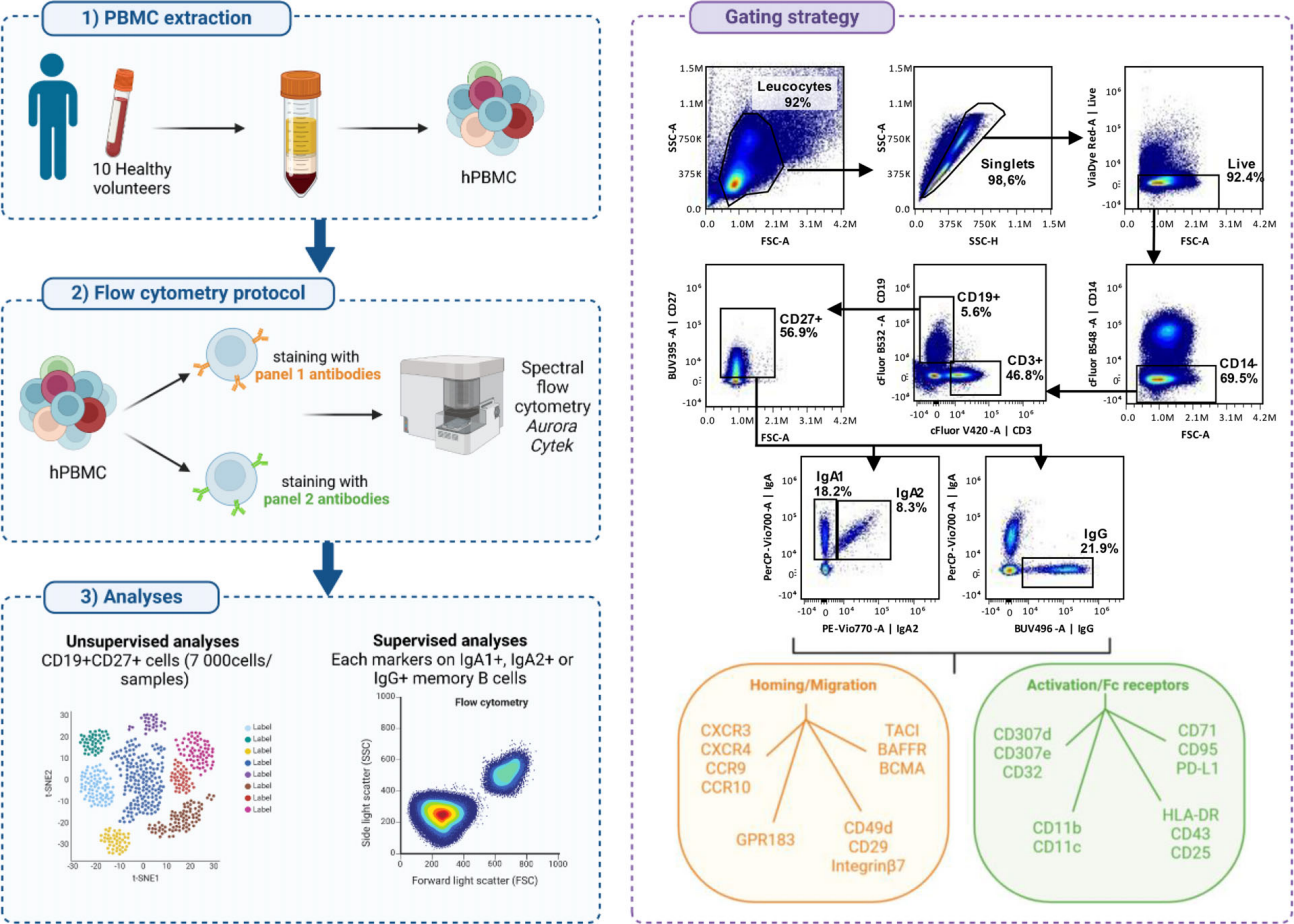
Method and gating strategy of flow cytometry. Schematic representation of the methods and the gating strategy used for the spectral flow cytometry analysis (realized on Biorender).

**FIGURE 2:**
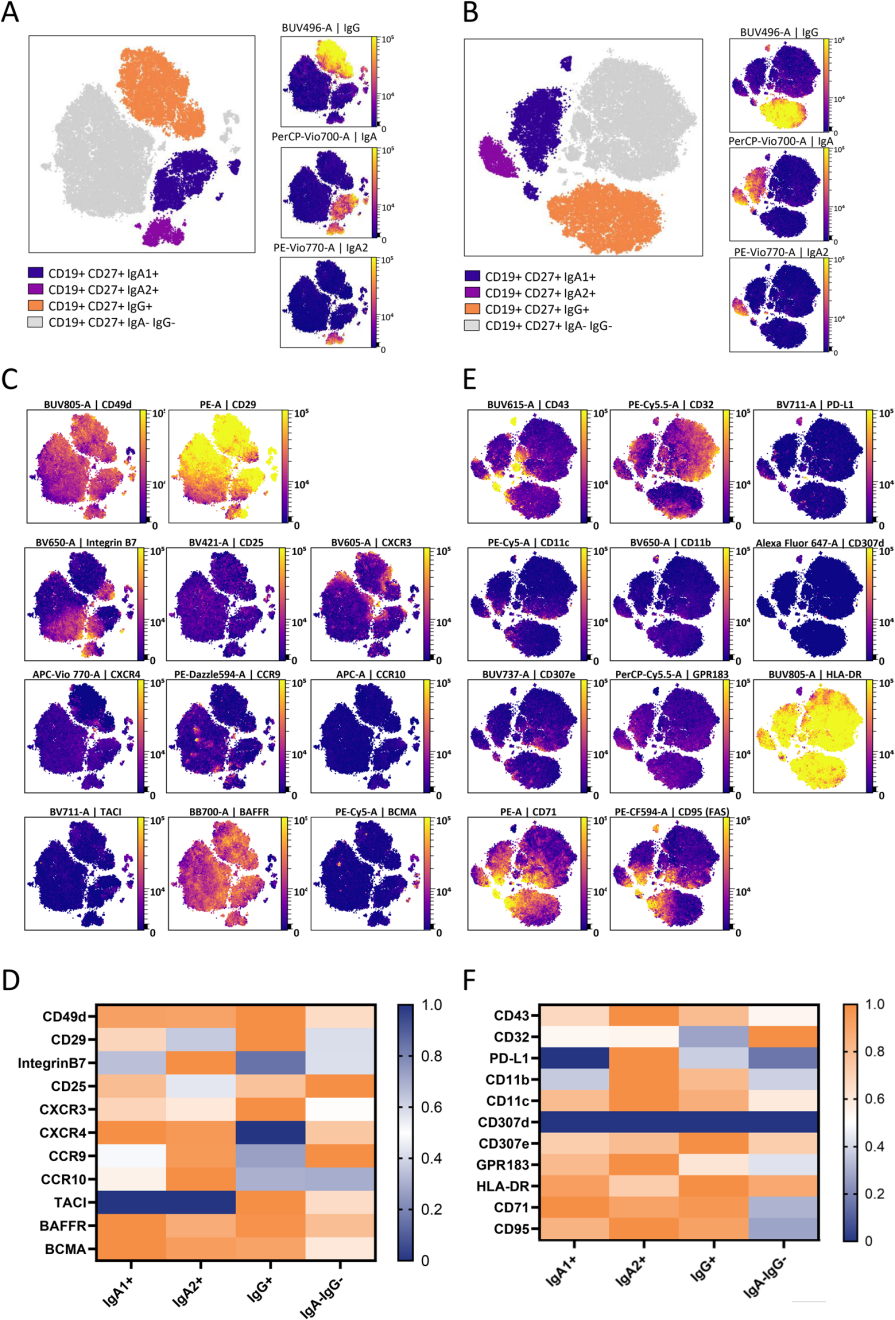
Unsupervised analysis of IgA1^+^, IgA2^+^, and IgG^+^ memory B cells. (A) T‐SNE CUDA projections of concatenated spectral cytometry data for memory B cells (CD19^+^ CD27^+^) of healthy volunteer samples labelled with panel 1. Cells were manually annotated after automatic clustering depending on IgA, IgA2, and IgG expression. (B) T‐SNE CUDA projections of concatenated spectral cytometry data for memory B cells (CD19^+^ CD27^+^) from healthy volunteer samples labelled with panel 2 antibodies. Cells were grouped and manually annotated after automatic clustering depending on IgA, IgA2, and IgG expression. (C) Relative expression of each analyzed marker from panel 1 (left panel) and panel 2 (right panel). (D) Heatmap representation showing the relative expression of each marker from panel 1 on IgA1^+^ memory B cells, IgA2^+^ memory B cells, and IgG^+^ memory B cells (upper panel) and from panel 2 (lower panel). (HV n = 10). (E) Relative expression of each of the analyzed markers from panel 2. (F) Heatmap representation showing the relative expression of each marker analyzed from panel 2 on IgA1^+^ memory B cells, IgA2^+^ memory B cells, IgG^+^ memory B cells, and IgA^−^IgG^−^ memory B cells. *n* = 10 healthy volunteers.

To better characterize IgA^+^ B cells, we first performed a spectral flow cytometry analysis on PBMCs from 10 healthy volunteers using 34 distinct markers divided into two panels (Figure 1). The first panel was designed to study migration and chemotaxis markers, and the second one focused on activation markers and Fc receptors (Table S1). Memory B cells were defined as CD19^+^CD27^+^ cells and subsampled to conduct an unsupervised clustering analysis (Figure 1; Figure S1A). Due to the limited number of plasmablasts and plasma cells detected (426 ± 453 cells per donor despite acquisition of 1 × 10^6^ PBMCs), analyses were restricted to the memory B cell compartment. This approach identified four cell clusters: IgA1^+^ memory B cells (corresponding to CD19^+^CD27^+^IgA^+^IgA2^−^IgG^−^ gating), IgA2^+^ memory B cells (CD19^+^CD27^+^IgA^+^IgA2^+^IgG^−^), IgG^+^ memory B cells (CD19^+^CD27^+^IgA^−^IgA2^−^IgG^+^), and IgA^−^IgG^−^ memory B cells (CD19^+^CD27^+^IgA^−^IgA2^−^IgG^−^) in panels 1 and 2 (Figure 2A,B). IgA1^+^, IgA2^+^, IgG^+^, and IgA^−^IgG^−^ memory B cells represented, respectively, 6.56 ± 2.64%, 2,15 ± 1.63%, 14.06 ± 4.61%, and 75.31 ± 7.89% of circulating memory B cells among the 10 healthy volunteers. Expression level (mean fluorescence intensity, MFI) of each marker was examined across these four clusters to obtain an overview of their phenotypic landscape.

Analysis of migration and chemotaxis‐related markers revealed both shared features and subset‐specific patterns across memory B cell populations. All clusters displayed high expression of CD49d (integrin α4 or ITGA4). CD29 (integrin β1 or ITGB1) was mostly expressed by IgA1^+^ and IgG^+^ memory B cells, whereas lower expression was found in IgA2^+^ and double‐negative memory B cells (Figure 2C,D), reflecting different use of the α4β1 integrin. Indeed, the combination of these two markers forms the cell adhesion integrin α4β1 (or VLA‐4), which plays a major role in cellular migration to the central nervous system and inflamed tissues [22]. By contrast, a low expression of integrin β7 was observed in all subclusters except for IgA2^+^ memory B cells (Figure 2C,D), supporting preferential formation of the gut‐homing α4β7 (LPAM‐1) integrin complex [22] in this population.

Chemokine receptor expression further distinguished memory B cell subsets. IgA1^+^ memory B cells exhibited a high expression of CXCR3 and CXCR4 and a moderate expression of CCR9 and CCR10 (Figure 2C,D). IgA2^+^ memory B cells preferentially expressed CXCR4, CCR9, and CCR10 and, to a lesser extent, CXCR3 (Figure 2C,D). The IgG^+^ memory B cell was characterized by a strong CXCR3 expression, but a weak expression of CXCR4, CCR9, and CCR10 (Figure 2C,D). Finally, IgA‐IgG^−^ memory B cells displayed high CCR9 expression, intermediate CXCR4 expression, and low levels of CXCR3 and CCR10 (Figure 2C,D). Furthermore, we observe significant expression disparities within each cluster for several markers: CD49d, CD29, Integrin β7, CXCR5, and CCR9, indicating significant heterogeneity in migratory functions within the same B cell subpopulation.

Concerning the BAFF/APRIL pathway involved in cell survival as well as in the B lymphocyte chemotaxis, all IgA1^+^, IgA2^+^, and IgG^+^ clusters highly express BAFFR (or TNFRSF13C or CD268) and BCMA (or TNFRSF17 or CD269), whereas TACI (or TNFRSF13B) expression appears to be specific to IgG^+^ and IgA^−^IgG^−^ memory B cells (Figure 2C,D). Taken together, these observations do not allow us to establish a specific expression signature for IgA+ memory B cells compared with IgG^+^ and IgA^−^IgG^−^ memory B cells.

Greater phenotypic disparities were observed among activation and regulation markers. PD‐L1 and CD11b were more strongly expressed by IgA2^+^ memory B cells compared with other clusters (Figure 2E,F). A strong expression of CD32 (Fc receptor of IgG FcγRII) was found in the double‐negative memory B cell cluster (Figure 2E,F). For the markers: CD307e (FcRL5), GPR183 (EBI2), HLA‐DR, CD71 (transferrin receptor TfR1), and CD95 (FAS), no difference was observed between the two IgA1^+^ and IgA2^+^ B cell clusters and the IgG^+^ memory B cell cluster; all three clusters strongly expressed these markers (Figure 2E,F). In contrast, double negative memory B cells expressed very low levels of GPR183, CD71, and CD95 (Figure 2E,F). Once again, a heterogeneous expression of several markers within the clusters was observed for CD43, CD32, CD71, and CD95. High and widespread expression of HLA‐DR was noted across all cell clusters (Figure 1E,F). Furthermore, strong co‐expression of CD95, CD71, and CD43 was observed in all clusters with HLA‐DR (Figure 2E,F). To note, high expression of CD95, associated with strong expression of HLA‐DR, has been described as a marker of a high degree of cell activation [23]. These observations indicated that within each subgroup, there are different states of activation. Furthermore, the activation mechanisms of IgA^+^ memory B cells and memory IgG^+^ B cells are broadly similar, unlike those of IgA^−^IgG^−^ memory B cells.

### 2.2 Supervised Analysis Revealed Differential Expression of Homing Markers and Chemokine/Cytokine Receptors

Following the initial unsupervised analysis on all markers expressed by memory B cells, we further looked at the frequencies and MFI for each marker using a supervised analysis. Among the 27 markers (excluding the eight population markers), eight markers displayed significantly different frequencies between IgA^+^ and IgG^+^ memory B cells. Ten additional markers showed comparable frequencies in IgA^+^ and IgG^+^ memory B cells, but were less frequent than in the IgA^−^IgG^−^ double‐negative subset.

**FIGURE 3:**
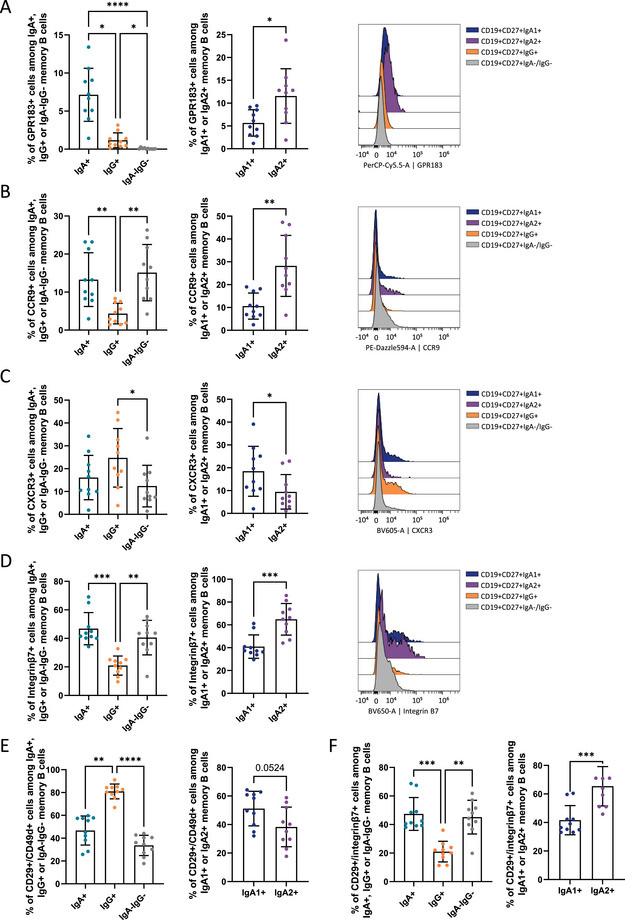
Markers of homing were differentially expressed on IgA^+^ and IgG^+^ memory B cells, with an increase of GPR183, CCR9, and integrin β7 and a decrease of CXCR3 expression in IgA^+^ B cells. (A) GPR183 expression and frequency of GPR183^+^ cells in IgA1^+^, IgA2^+^, IgG^+^, and IgA^−^IgG^−^ memory B cells (CD19^+^/CD27^+^). (B) CCR9 expression and frequency of CCR9^+^ cells in IgA1^+^, IgA2^+^, IgG^+^, and IgA^−^IgG^−^ memory B cells (CD19^+^/CD27^+^). (C) CXCR3 expression and frequency of CXCR3^+^ cells in IgA1^+^, IgA2^+^, IgG^+^, and IgA^−^IgG^−^ memory B cells (CD19^+^/CD27^+^). (D) Integrin β7 expression and frequency of Integrin β7+ cells in IgA1^+^, IgA2^+^, IgG^+^, and IgA^−^IgG^−^ memory B cells (CD19^+^/CD27^+^). (E) α4β1^+^ (CD49D^+^/CD29^+^) cell frequency in IgA^+^, IgA1^+^, IgA2^+^, IgG^+^ and IgA^−^IgG^−^ memory B cells (CD19^+^/CD27^+^). (F) α4β7^+^ (CD49D^+^/CD29^+^) cell frequency in IgA^+^, IgA1^+^, IgA2^+^, IgG^+^ and IgA^−^IgG^−^ memory B cells (CD19^+^/CD27^+^). *n* = 10 healthy volunteers. The *p‐*values were calculated using the Kruskal–Wallis test with Dunn's posttest (*p < 0.05; **p < 0.01; ***p < 0.001; ****p < 0.0001).

Regarding the expression of homing markers (GPR183, integrins, CXCR3, CXCR4, CCR9, and CCR10), no significant differences were observed for CXCR4 and CCR10 expressions among IgA^+^ and IgG^+^ memory B cells; both receptors were not expressed in the double‐negative population (Figure S2A; FigureS2B). In contrast, clear divergence was observed for other homing receptors. GPR183 was notably enriched in IgA^+^ memory B cells compared with IgG^+^ and double‐negative subsets, with the highest expression in IgA2^+^ memory B cells compared with IgA1^+^ memory B cells (Figure 3A, frequencies and MFI). Moreover, CCR9 expression was significantly higher on IgA^+^ and double‐negative memory B cell subsets than on IgG^+^ cells, again with a pronounced enrichment in the IgA2^+^ memory B cell subset (Figure 3B, frequencies and MFI). No significant differences between IgA^+^ and IgG^+^ memory B cells were observed concerning the expression of the membrane receptor CXCR3. However, this receptor is preferentially expressed by IgG^+^ memory B cells compared with the double‐negative subset. Within the IgA compartment, IgA1^+^ memory B cells displayed higher CXCR3^+^ frequency and MFI than IgA2^+^ memory B cells (Figure 3C).

Integrinβ7^+^ cell frequency was higher in the IgA^+^ and double‐negative memory B cell subsets compared with IgG^+^ cells, with IgA2^+^ cells exhibiting higher expression than IgA1^+^ cells (Figure 3D, frequencies and MFI). The cell adhesion integrin α4β1 (VLA4) was mainly expressed by IgG^+^ memory B cells (Figure 3E); meanwhile, the cell adhesion integrin α4β7 (LPAM‐1) was mostly expressed by IgA^+^ and IgA^−^IgG^−^ memory B cells (Figure 3F), with a higher frequency of α4β7 in IgA2^+^ memory B cells. These expressions suggest a more pronounced gut homing/origin for IgA^+^ memory B cells and lymph node and inflamed organ migration markers for IgG^+^ memory B cells. These findings define a distinct homing receptor signature of IgA^+^ memory B cells, especially the IgA2^+^ subset, characterized by a higher expression of GPR183, CCR9, and integrinβ7 and a reduced expression of CXCR3.

Expression of the three receptors of BAFF/APRIL: TACI, BAFFR, and BCMA (Figure S2C–F) was assessed across memory B cell subsets. No significant differences in expression frequency were found for any of these receptors between IgA^+^ and IgG^+^ memory B cells, indicating that those two subsets appeared to be affected by those cytokines in the same way. In contrast, the IgA^−^IgG^−^ subset exhibited significantly lower expression frequencies of both TACI and BCMA markers compared with IgA^+^ and IgG^+^ memory B cells. This may reflect a diminished engagement of the BAFF/APRIL‐dependent signalling pathway in double‐negative memory B cells.

**FIGURE 4:**
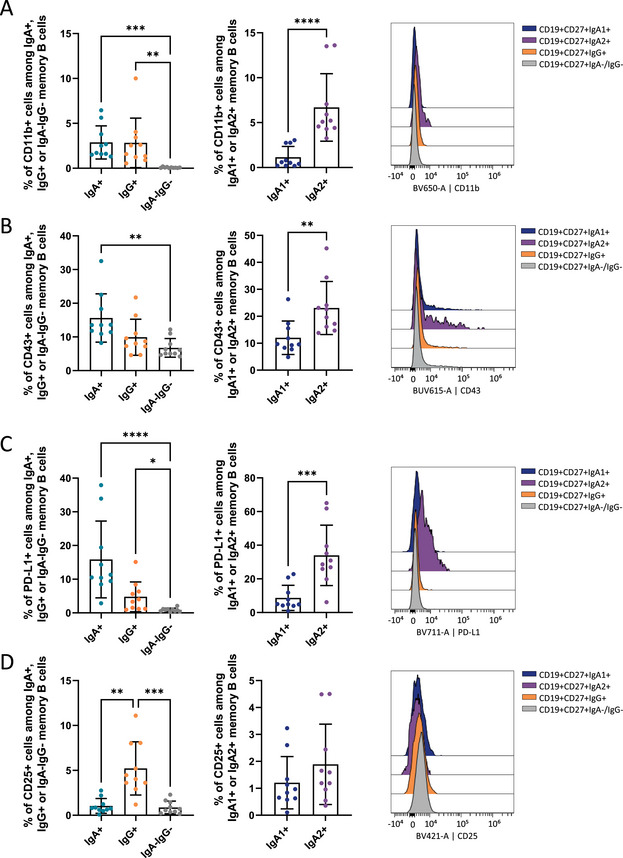
IgA2^+^ memory B cells expressed more PD‐L1, CD11b, and CD43, whereas IgG^+^ memory B cells expressed more CD25. (A) CD11b expression and frequency of CD11b^+^ cells in IgA1^+^, IgA2^+^, IgG^+^, and IgA^−^IgG^−^ memory B cells (CD19^+^/CD27^+^). (B) CD43 expression and frequency of CD43^+^ cells in IgA1^+^, IgA2^+^, IgG^+^, and IgA^−^IgG^−^ memory B cells (CD19^+^/CD27^+^). (C) PD‐L1 expression and frequency of PD‐L1^+^ cells in IgA1^+^, IgA2^+^ IgG^+^, and IgA^−^IgG^−^ memory B cells (CD19^+^/CD27^+^). (D) CD25 expression and frequency of CD25^+^ cells in IgA1^+^, IgA2^+^, IgG^+^, and IgA^−^IgG^−^ memory B cells (CD19^+^/CD27^+^). *n* = 10 healthy volunteers. The *p*‐values were calculated using the Kruskal–Wallis test with Dunn's posttest (*p < 0.05; **p < 0.01; ***p < 0.001; ****p < 0.0001).

The second panel mainly focused on activation markers and Fc receptors. We observed a higher frequency of CD11b^+^ (integrin αM or ITGAM) cells in IgA2^+^ memory B cells (Figure 4A), while IgA^−^IgG^−^ memory B cells did not express this marker. Within the IgA compartment, IgA2^+^ memory B cells exhibited a higher frequency of CD11b^+^ cells than IgA2^+^ memory B cells, associated with a higher CD11b MFI. No significant differences in CD43 expression were observed between IgA^+^ and IgG^+^ memory B cells (Figure 4B). However, IgA^+^ cells have a higher expression (MFI and frequency) of this marker compared with the double‐negative population, with the strongest expression in the IgA2^+^ subset. Both IgA^+^ and IgG^+^ memory B cells expressed higher levels of PD‐L1 than IgA^−^IgG^−^ memory B cells, with a clear enrichment in the IgA2^+^ compartment (Figure 4C).

No significant differences in either frequency or expression level were detected between IgA^+^ and IgG^+^ memory B cells for CD32 (Figure S3A), CD11c (integrin αX or ITGAX) (Figure S3B), CD307e (or FcRL5) (Figure S3C), CD307d (or FcRL4) (Figure S3D), HLA‐DR (Figure S4A), CD71 (Figure S4B), and CD95 (Figure S4C). Despite the overall similarity in activation marker profile between IgA^+^ and IgG^+^ memory B cells, these analyses revealed a clear, distinct phenotype for the double‐negative memory B cell population. This cluster displayed significantly higher CD32 expression (frequency and MFI), together with reduced expression frequencies of CD11c, CD307d, CD71, and CD95. This pattern in double‐negative cells is consistent with a specific activation mechanism distinct from IgA^+^ and IgG^+^ memory B cells.

Finally, analysis of cytokine receptor revealed significant lower frequency of CD25 (IL2RA)^+^ cells within IgA^+^ and IgA^−^IgG^−^ memory B cells compared with IgG^+^ memory B cells (Figure 4D), with no detectable differences between IgA1^+^ and IgA2^+^ subsets.

### 2.3 Transcriptomic Analysis of Memory B Cells Showed Markers Associated With IgA^+^ and IgG^+^ Memory B Cells, Different From IgM^+^ Memory B Cells

**FIGURE 5:**
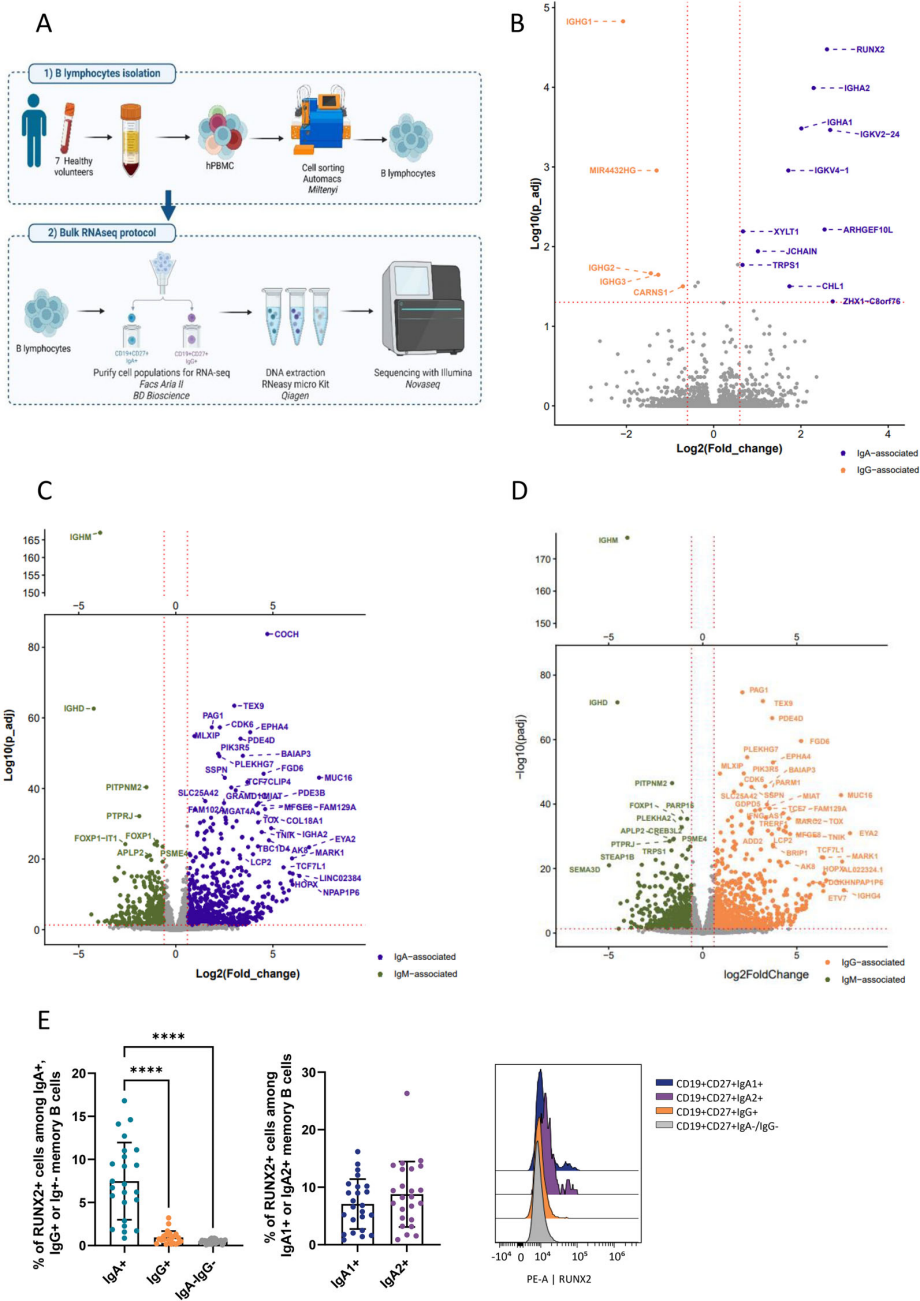
Transcriptomic analysis of sorted IgA^+^  IgG^+^ and IgM^+^ memory B cells. (A) Schematic representation of the methods for bulk RNA sequencing analysis (realized on Biorender). (B) Volcano plot of differentially expressed genes between IgA^+^ and IgG^+^ memory B cells. *n* = 7 healthy volunteers. (C) Volcano plot of differentially expressed genes between IgA^+^ and IgM^+^ memory B cells. *n* = 7 healthy volunteers. (D) Volcano plot of differentially expressed genes between IgG^+^ and IgM^+^ memory B cells. *n* = 7 healthy volunteers. (E) RUNX2 protein expression and frequency of RUNX2^+^ cells in IgA1^+^, IgA2^+^, IgG^+^, and IgA^−^IgG^−^ memory B cells (CD19^+^/CD27^+^) assessed by flow cytometry. n = 24 healthy volunteers. The *p*‐values were calculated using the Kruskal–Wallis test with Dunn's posttest (*p < 0.05; **p < 0.01; ***p < 0.001; ****p < 0.0001).

To complete this analysis, we performed a bulk RNA sequencing analysis on sorted memory B cells (Figure 5A). This analysis revealed a few differentially expressed genes between IgA^+^ and IgG^+^ memory B cells (Figure 5B; Table S2, *p* adjusted <0.05). As expected, *IGHA1* and *IGHA2* mRNA were upregulated in IgA+ memory B cells, and *IGHG1*, *IGHG2*, and *IGHG3* mRNA were expressed in IgG^+^ memory B cells. *RUNX2*, *ARHGEF10L*, *TRPS1*, *XYLT1*, *CHL1*, and *ZHX1‐C8orf76* mRNA were highly expressed in IgA^+^ B cells, while the *miR4432* and *CARNS1* mRNA were upregulated in IgG^+^ memory B cells. Concerning IgM^+^ memory B cells, their transcriptomic signature was highly different from IgA^+^ and IgG^+^ memory cells (Figure S5, principal component analysis). Comparing IgM^+^ and IgA^+^ memory cells (Figure 5C: volcano plot and Table S3: list of genes), five genes were significantly highly upregulated in IgM^+^ cells and 34 genes in IgA^+^ cells. Comparing IgM^+^ and IgG^+^ memory cells (Figure 5D; Table S4), 11 genes were highly upregulated in IgM^+^ cells and 35 genes in IgG^+^ cells. Interestingly, among those genes, 5 genes were commonly upregulated in IgM^+^ memory B cells compared with both IgA^+^ and IgG^+^ memory B cells: *PITPNM2, PTPRJ, FOXP1, APLP2*, and *PSME4*. Inversely, 24 common genes coding mainly transcription factors and kinases were upregulated in IgA^+^ and IgG^+^ memory B cells compared with IgM^+^ cells: *AK8, CDK6, COCH, EPHA4, EYA2, FAM129A, FGD6, HOPX, LCP2, MARK1, MIAT, MLXIP, MUC16, NPAP1P6, PAG1, PDE4D, PIK3R5, PLEKHG7, SLC25A42, SSPN, TCF7L1, TEX9, TNIK*, and *TOX*. So the transcriptomic signature of class switch memory B cells seems to be shared between IgA^+^ and IgG^+^ cells, distinct from IgM^+^ memory B cells.

A flow cytometry analysis was conducted to confirm the expression of RUNX2 on memory IgA^+^ B cells at the protein level. Consistent with transcriptomic analyses, a higher frequency of RUNX2 expression was observed in IgA^+^ memory B cells (Figure 5E). The expression of this marker appeared to be specific to this population without a difference between IgA1^+^ and IgA2^+^ memory B cells (Figure 5E).

## 3 Discussion

IgA is by far the most produced antibody in the human body and is mainly considered a passive immune component as it provides immune exclusion of pathogens in the intestinal tract. However, different studies showed that IgA can also have an active role in immunity by its capacity to modulate cytokine production [24, 25] and exacerbate inflammation in host defense [26]. Although the functions of IgA at mucosal surfaces are increasingly studied and recognized [27]^,^ circulating IgA and IgA‐producing B lymphocytes remain relatively underexplored. Given the various roles of IgA in immunity, it is essential to further investigate IgA^+^ B lymphocytes as they have important functions in immune regulation [25]. Here, using a large panel of antibodies in spectral flow cytometry and transcriptomic analysis, we deeply compared IgA^+^ memory B cells to IgG^+^ memory B cells.

In this study, we identify preferential expression of mucosal homing markers in blood IgA^+^ memory B cells and, more specifically, in IgA2^+^ memory B cells. IgA2^+^ memory B cells exhibited a higher frequency and level of expression of CCR9 and CCR10 than IgG^+^ memory B cells, two chemokine receptors playing a major role in the trafficking of lymphocytes to the gut. CCR9 binds to the chemokine CCL25 [28, 29, 30]. Our findings regarding CCR10 are consistent with current literature, reporting 70% of IgA‐producing cells (plasmablasts and plasma cells expressing CCR10 [31]. CCR10 is involved in multiple functions of mucosal immunity, not only maintaining long‐lived IgA^+^ plasma cells and IgA^+^ memory B cells in the intestine, but also allowing IgA^+^ plasma cell migration to the intestine during inflammation through its binding to CCL28 [32]. CCR10 is also the receptor of CCL27, which is mostly found in human epidermal keratinocytes [29, 30, 31, 32, 33]. The expression of CCR9 and CCR10 on circulating IgA2^+^ memory B cells reflects their dissemination in the different mucosal sites.

At the protein level, CXCR3 expression was similar between IgA^+^ and IgG^+^ memory B cells. CXCR4 expression was increased in IgG^+^ memory B cells compared with IgA^+^ memory B cells. By contrast, double‐negative memory B cells expressed CXCR4 at an extremely low level. The balance of expression between these two chemokine receptors is important for B cell development and determines the migratory behavior of B cells during immune responses [34, 35]. CXCR4 promotes retention in lymphoid tissues, whereas CXCR3 permits migration to inflammation sites or germinal centers [36].

Interestingly, we found a higher expression of integrin α4β7 (LPAM‐1) by IgA^+^ memory B cells compared with IgG^+^ B cells. This cell adhesion integrin is important for antibody‐secreting cell trafficking to the gut through its interaction with MAdCAM‐1, mainly expressed by intestinal endothelial cells. B and T lymphocytes employ LPAM‐1 to migrate to the intestine under homeostatic conditions and chronic inflammation [37]. In addition, the expression of the cell adhesion integrin α4β1 (VLA‐4 or CD49d/CD29) by IgA^+^ memory B cells is important but lower than that of IgG^+^ memory B cells. This integrin plays an important role in B lymphocyte development and function, as it can provide a costimulatory signal contributing to lymphocyte activation [38, 39]. VLA‐4 expressed by B cells interacts with VCAM‐1 expressed by stromal cells, maintaining close contact between developing B cells and stromal cells and providing essential growth factor and signaling [40]. VLA4 expression by IgA^+^ memory B cells suggests that it could be crucial for their long‐term survival [41], their activation and interaction with the microenvironment, but also their migration to mucosal tissues. Our study also demonstrated a higher expression of GPR183 (EBI2) in IgA^+^ memory B cells. GPR183 binds to oxysterol 7a,25‐dihydroxycholesterol and is highly expressed on naive B cells, required to permit their entry into inflamed lymph nodes in coordination with other receptors [42]. Thus, IgA^+^ memory B cells, through the differential expression of these markers, appear to exhibit migratory capabilities that differ from those of IgG^+^ B cells. They appear to exhibit expression of factors involved in migration mechanisms, particularly toward mucosal tissues such as the gut.

The investigation of the BAFF/APRIL pathway through the study of BAFF and APRIL receptor expression did not reveal major differences between IgA^+^ and IgG^+^ memory B cells, except for BCMA, which was more expressed by IgA^+^ memory B cells. BCMA is a ligand for APRIL, which is principally produced in the epithelium of the tonsil. This interaction seems necessary for IgA production as APRIL knockout mice exhibit reduced IgA levels [43]. Overall, we observed an unsurprisingly strong expression of BAFFR both in IgA^+^ and IgG^+^ memory B cells, as this pathway is crucial for general B cell survival and maturation [44]. In addition, BAFF signalling, in contrast to APRIL signalling, plays a key role in the chemotaxis of memory B cells within lymphoid tissues through its binding to the chemokine CXCL13 [45]. IgA^+^ memory B cells seem to be more dependent on BAFF/APRIL signalling for their survival and their production than IgG^+^ memory B cells.

Transcriptomic analysis reveals that the transcription factor RUNX2 was highly expressed by IgA^+^ memory B cells, which we confirmed at the protein level. Our data confirm a previous study from Berkowska et al. [46] showing RUNX2 as a marker of IgA+ memory B cells. This transcription factor is implicated in early B cell development and migration from the bone marrow to peripheral tissue [47]. RUNX2‐RUNX3 deficiency in mice affects IgA class switch and IgA production [48]. Among the gene targets of RUNX2, receptors of chemokines and integrins, such as CCR2, CCR4, CCR5, CCR7, CXCR4, ITGAX, and ITGAM, are modulated by the expression of RUNX2 [49, 50, 51].

Concerning activation of B cells, IgA2^+^ memory B cells exhibit a higher expression frequency of CD43 (or sialophorin). In B cells, CD43 is typically expressed on early B cell precursors and on plasma cells [52]–[53]. Studies have shown that CD43 dysregulation affects B cell development, proliferation, and survival [54, 55, 56]. Our study reveals a lower expression frequency of the interleukin‐2 receptor alpha chain (IL‐2Rα or CD25 on IgA^+^ memory B cells compared with IgG^+^ memory B cells. The expression of CD25 by B cells is necessary for their survival as IL‐2 signaling enhances proliferation and STAT3 activation [57]. B lymphocytes expressing CD25 are highly efficient antigen‐presenting cells; they also tend to express costimulatory molecules, allowing T‐cell activation [57]. CD25 also seems to regulate BCR signaling, preventing hyperactivation and autoimmunity [58]. The absence of differential expression between IgA^+^ and IgG^+^ memory B lymphocytes regarding activation markers, including Fc receptors, suggests IgA^+^ memory B cells share many activation pathways with IgG^+^ memory B cells.

Markers associated with immune regulation were also different between IgA^+^ and IgG^+^ memory B cells. Indeed, we found a higher frequency of IgA2^+^ memory B cells expressing CD11b (or ITGAM). It plays various functional roles, such as the negative regulation of the BCR signalling that helps prevent hyperactivation of B cells [59, 60] and modulates antibody production by regulating plasma cell activity [60]. Moreover, the higher expression of PD‐L1 observed on those cells reinforced this hypothesis. The expression of PD‐L1 is associated with a regulatory B cell phenotype. It suppresses T‐cell activation and proliferation, and this leads to a cytokine expression modulation [60].

Taken together, our data highlight specific markers of migration to mucosa and regulatory markers expressed by IgA^+^ memory B cells compared with IgG^+^ memory B cells and indicate that IgA^+^ and IgG^+^ memory B cell subsets share most of the activation markers.

## 4 Materials and Methods

### 4.1 Blood Samples

Blood from healthy donors was collected at the Department of Neurology, Nantes Hospital, France. The study was approved by the ethics committee (project ABM PFS13‐003) and conducted in agreement with the Helsinki Declaration. Fresh peripheral blood mononuclear cells (PBMCs) were isolated using a lymphocyte isolation gradient (Eurobio).

### 4.2 Flow Cytometry Analysis

Flow cytometry analyses adhered to the “Guidelines for the use of flow cytometry and cell sorting in immunological studies” [61]. All the antibodies are listed in Table S1. Dead cells were excluded from the analysis using the ViaDye Red staining (ThermoFisher) for 20 min at room temperature in phosphate buffer saline solution (PBS). Then, surface markers were analyzed by staining PBMC in three steps for each panel. First, for panel 1, cells were stained for 10 min at 4°C in running buffer (PBS with 5% bovine serum albumin (BSA) and 2 mM EDTA) with the following conjugated antibodies: anti‐CD138 coupled with V450; anti‐CD38 cFluor R720, anti‐CD199 PE‐Dazzle594, anti‐CCR10 APC, anti‐CD183 BV605, anti‐IgA PerCP‐Vio700, anti‐IgA2 PE‐Vio770, anti‐IgG BUV 496. Then, cells were stained for 10 min at 4°C in running buffer with: anti‐Integrin β7 BV650, anti‐CD29 PE, anti‐CD267 BV711, anti‐CD269 PE‐Cy5, anti‐CD25 BV421, anti‐CD268 BB700, anti‐CD49d BUV 805. All antibody information is available in Table S1.

For panel 2, cells were stained for 10 min at 4°C in running buffer with: anti‐CD307d Alexa647, anti‐CD307e BUV737, anti‐CD274 BV711, anti‐GPR183 PerCP‐Cy5.5, anti‐CD138 V450; anti‐CD38 cFluor R720, anti‐IgA PerCP‐Vio700, anti‐IgA2 PE‐Vio770, and anti‐IgG BUV 496. Then, cells were stained for 10 min at 4°C in running buffer with anti‐CD11b BV650, anti‐CD71 PE, anti‐CD95, anti‐CD43 BUV615, anti‐CD11c PE‐Cy5, anti‐CD32 PE‐Cy5.5, anti‐HLA‐DR BUV805.

Finally, to identify the B cell population, cells were stained for each panel, for 20 min at 4°C in running buffer with: anti‐CD20 cFluor V547 (Cytek Biosciences; clone 2H7), anti‐CD19 cFluor B532 (Cytek Biosciences; clone H1B19), anti‐CD3 cFluor V420 (Cytek Biosciences; clone SK7), anti‐CD14 cFluor B548 (Cytek Biosciences; clone 63D3), anti‐CD21 BUV563 (BD Optibuid; clone B‐ly4), and anti‐CD27 BUV395 (BD; clone L128). After staining, cells were fixed for 30 min at 4°C with a fixation buffer (eBioscience, Invitrogen, ThermoFischer Scientific) and washed with a permeabilization buffer (Permeabilization Buffer, eBioscience, Invitrogen, ThermoFischer Scientific). Stained cells were analyzed using a Cytek Aurora spectral Flow cytometer. Data analysis was performed using OMIQ software (Dotmatics, www.dotmatics.com) according to guidelines for the use of flow cytometry. After data compensation, the same gating strategy was applied for each individual to remove debris, doublets, and dead cells and to define the B cell population.

Second analysis with RUNX2 was performed using the same protocol on PBMC from 24 healthy volunteers with the following conjugated antibodies: anti‐CD20 cFluor V547 (Cytek Biosciences; clone 2H7), anti‐CD19 cFluor B532 (Cytek Biosciences; clone H1B19), anti‐CD3 cFluor V420 (Cytek Biosciences; clone SK7), anti‐CD14 cFluor B548 (Cytek Biosciences; clone 63D3), anti‐CD21 BUV563 (BD Optibuid; clone B‐ly4) and anti‐CD27 BUV395 (BD; clone L128), anti‐IgA PerCP‐Vio700 (Miltenyi; clone IS11‐8^E^10), anti‐IgA2 PE‐Vio770 (Miltenyi; clone IS11‐21^E^11), anti‐IgG BUV 496 (BD Optibuild; clone REA649), anti‐IgM BB700 (BD Optibuild; clone UCH‐B1). Then, cells were stained for 30 min at room temperature in permeabilization buffer with RUNX2 PE (Ozyme; clone D1L7F).

### 4.3 Memory B Cell Sorting and Bulk RNA Sequencing Analysis

For bulk RNA sequencing analysis, IgA^+^ and IgG^+^ memory B‐cells were sorted from frozen PBMCs. Before the sorting, a purification of B‐cells was performed using the B‐cell isolation kit II (Miltenyi Biotec) following the manufacturer's recommendations. B‐cells were then stained with the following anti‐human antibodies: anti‐IgA PE‐Vio770 and anti‐IgG APC (Miltenyi Biotec), anti‐IgM PE, anti‐CD19 FITC, and anti‐CD27 BV450 (BD Biosciences). Memory B‐cells were defined as CD19^+^CD27^+^ B‐cells, and a sorting of IgA^+^, IgG^+^, or IgM^+^ memory B‐cells was performed using a cell sorter (FACSAria II, BD Biosciences). Purity of cell sorting was >97.6%. Dead cells were excluded using Live/Dead AmCyan staining (Invitrogen). Cells sorted were then stored at −80°C in RLT buffer (Qiagen). Total mRNA was purified using the RNeasy Micro Kit (Qiagen). The RNA integrity and quantity were assessed using Agilent 2100 Bioanalyzer (Agilent Technologies). Total mRNA was then stored at −80°C until its use for the preparation of the sequencing library. cDNA libraries were obtained using SMARTer Stranded Total RNA‐Seq Kit v2—Pico Input Mammalian (Takara Bio), according to the manufacturer's instructions. RNAseq libraries were then sequenced with the NovaSeq Instrument (Illumina). Libraries preparation and sequencing were performed by the Next Generation Sequencing Platform (Institut Curie, Paris, France). Quality of sequencing data was monitored by FastQC. Low‐quality reads and bases were removed using Cutadapt v1.18. Reads were mapped to the human genome (Hg38) using the Star aligner, and a raw gene count matrix was obtained using the FeatureCount algorithm. Differential gene expression was performed using R software (v 3.6.3). Genes with the lowest variability were filtered out using HTSfilter, and differential expression between them was performed using the DESeq2 package. Genes were declared differentially expressed with a false discovery rate <5% and fold change ≥0.5 in IgA^+^ or IgG^+^ memory B‐cells. Hierarchical clustering was generated using the Ward method.

### 4.4 Statistical Analyses

Group comparisons were performed using the nonparametric Mann–Whitney test and Kruskal–Wallis with Dunn's posttest. The following *p*‐values were considered statistically significant: *p < 0.05; **p < 0.01; ***p < 0.001; ****p < 0.0001. Analyses were performed using GraphPad Prism v.10 (GraphPad Software).

## Author Contributions

**Louise Le Gal**: formal analysis, investigation, visualization, writing – original draft, and writing – review and editing. **Léo Boussamet**: formal analysis, investigation, visualization, and writing – review. **Alexandra Garcia**: formal analysis, investigation, visualization, validation, and writing – review. **Jérémy Morille**: formal analysis, investigation. **Emilie Dugast**: formal analysis, investigation. **Arnaud Nicot**: writing – review. **David‐Axel Laplaud**: Resources, supervision, and writing – review. **Laureline Berthelot**: conceptualization, supervision, validation, funding acquisition, project administration, writing – review and editing.

## Funding

The authors have nothing to report.

## Ethics Statement

This study is the control part of the project ABM PFS13‐003, Collection sclérose en plaques, which was reviewed and approved by the institutional ethics committee of Agence de la Biomédecine. All donors provided written informed consent in compliance with the Declaration of Helsinki.

## Conflicts of Interest

The authors declare no conflicts of interest.

## Supporting information




**Supporting File 1**: eji70191‐sup‐0001‐figuresS1‐S5.pdf.

**Supporting File 2**: eji70191‐sup‐0002‐tableS1.xlsx.

**Supporting File 3**: eji70191‐sup‐0003‐tableS2.xlsx.

**Supporting File 4**: eji70191‐sup‐0004‐tableS3.xlsx.

**Supporting File 5**: eji70191‐sup‐0005‐tableS4.xlsx.

## Data Availability

All analyses of flow cytometry that support the findings of this study are included in the paper and in the Supporting Information files. For raw data (FSC files) request, demand should be emailed to laureline.berthelot@inserm.fr. The transcriptomic data set generated and analyzed for the current study is available in the European read archive repository (https://www.ebi.ac.uk/ena/browser/home) under the accession number PRJEB87826.
