# Supplementary material for: Human Blood IgA+ Memory B Cells Differ From IgG+ B Cells by Expressing Gut‐Homing and Regulatory Markers
Source: Eur J Immunol. 2026 Apr 26;56:e70191. doi: 10.1002/eji.70191 (PMC13111729; doi:10.1002/eji.70191)
Supplement: Supplementary file 1 — Supporting File 1: eji70191‐sup‐0001‐figuresS1‐S5.pdf. [file EJI-56-e70191-s003.pdf]

Supplementary Figure 1

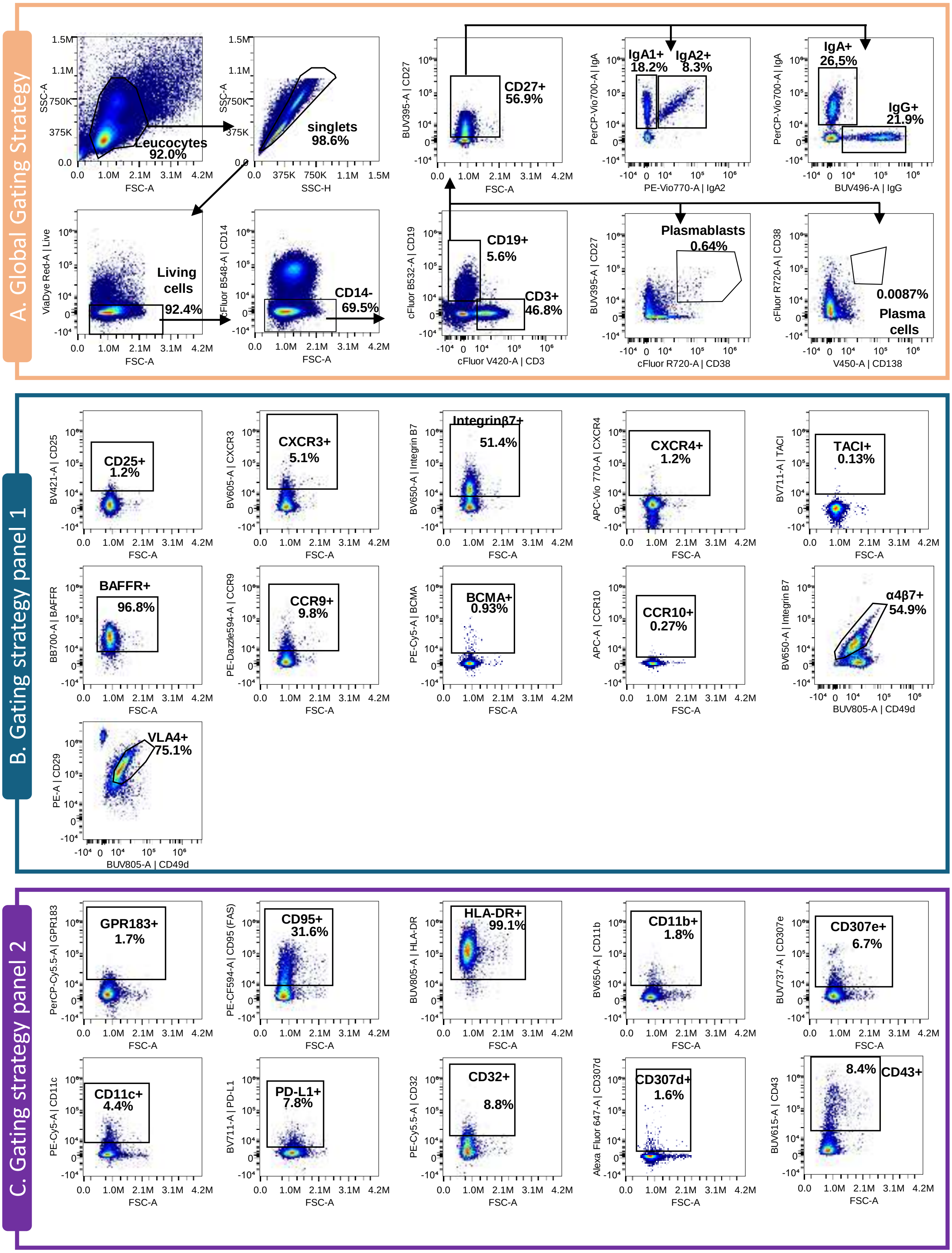

Supplementary Figure 2

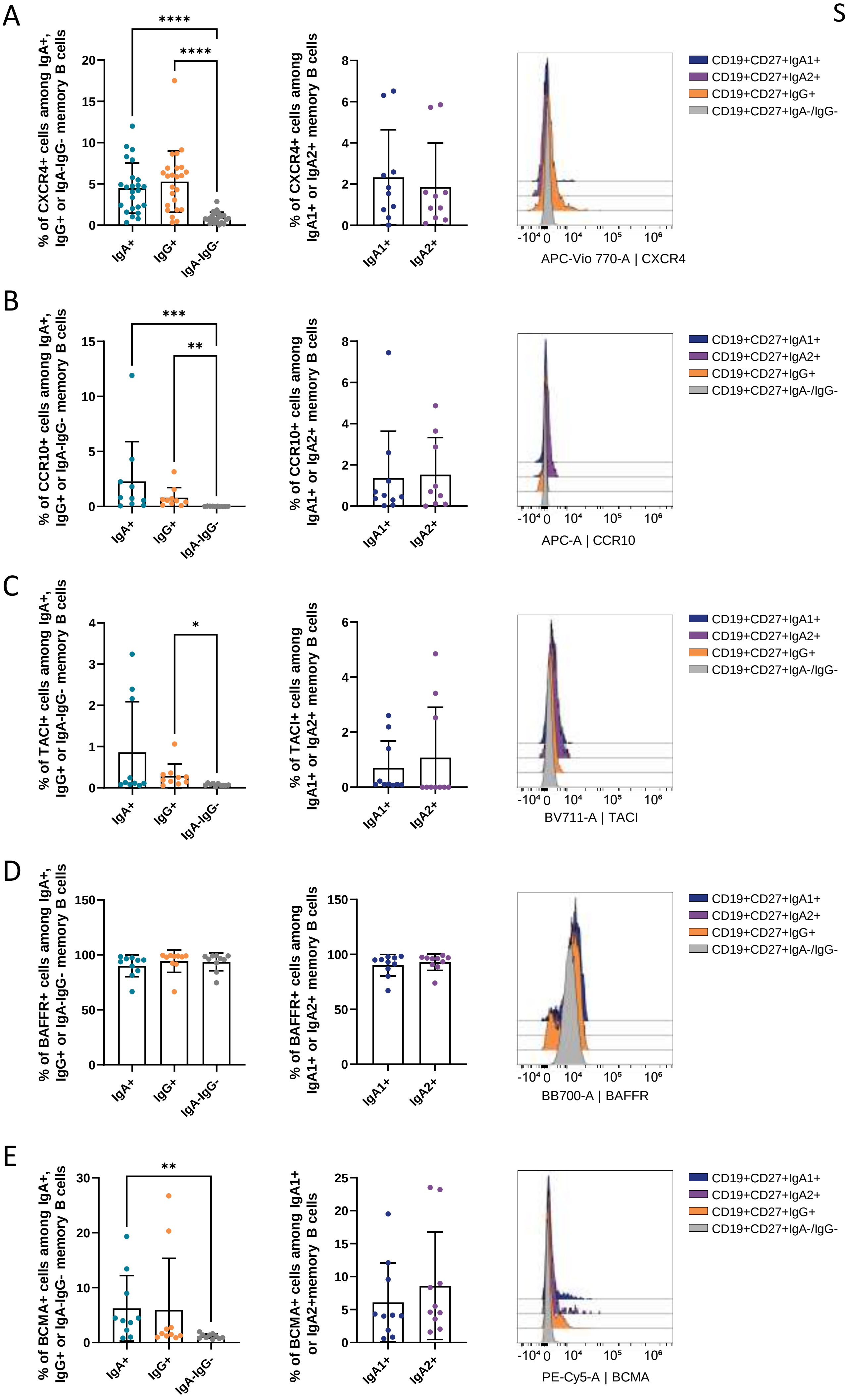

Supplementary Figure 3

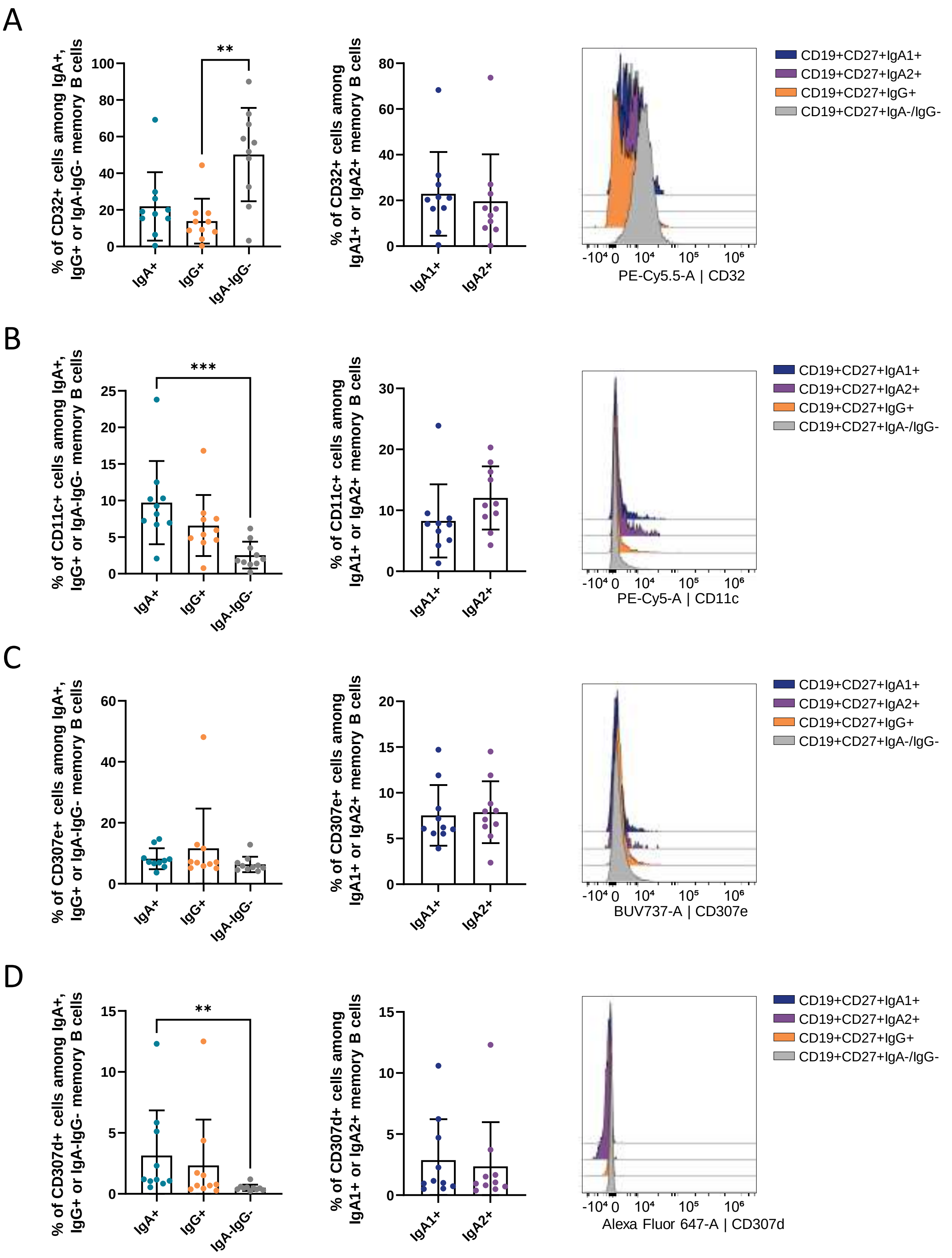

Supplementary Figure 4

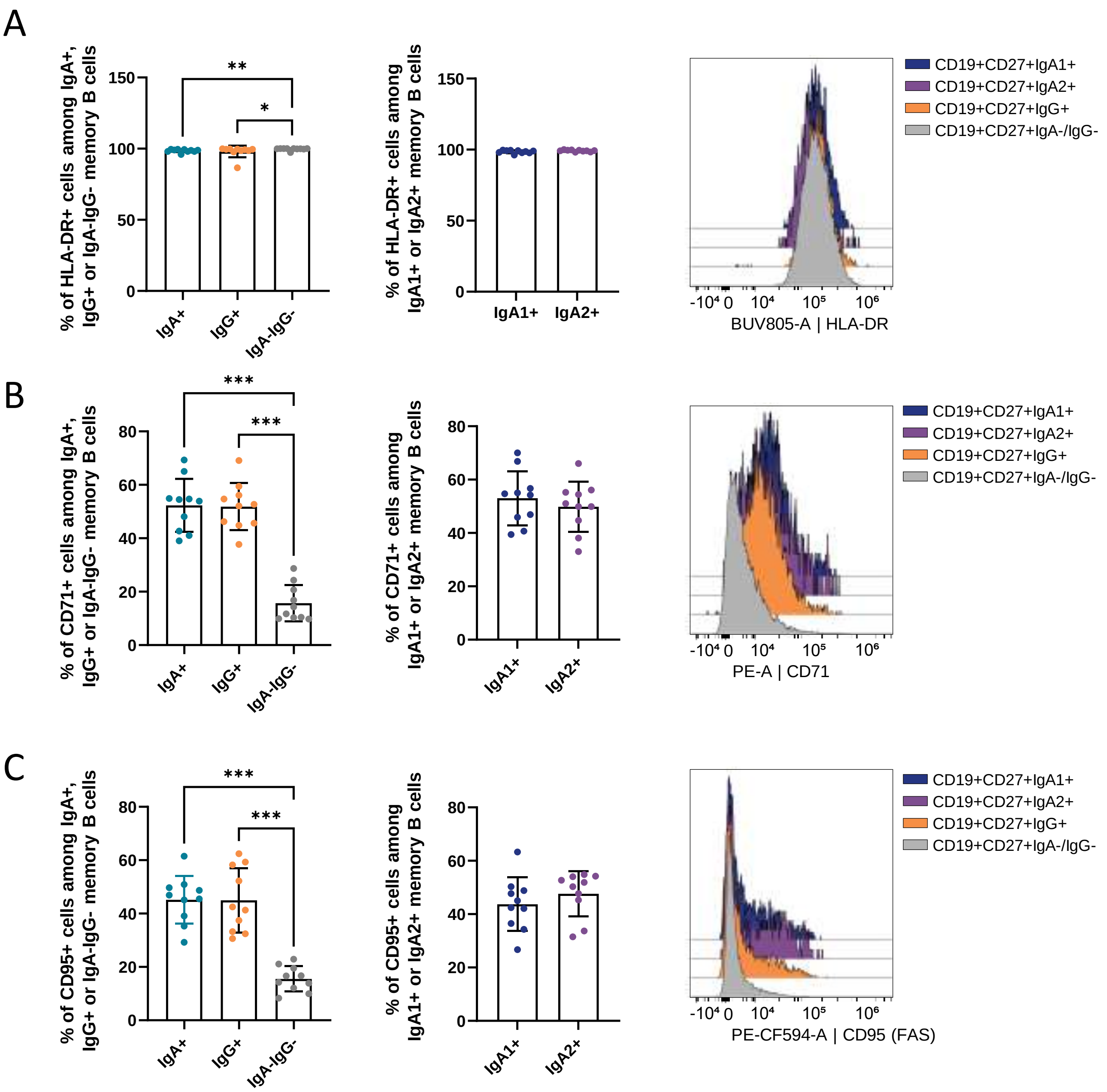

Supplementary Figure 5

A

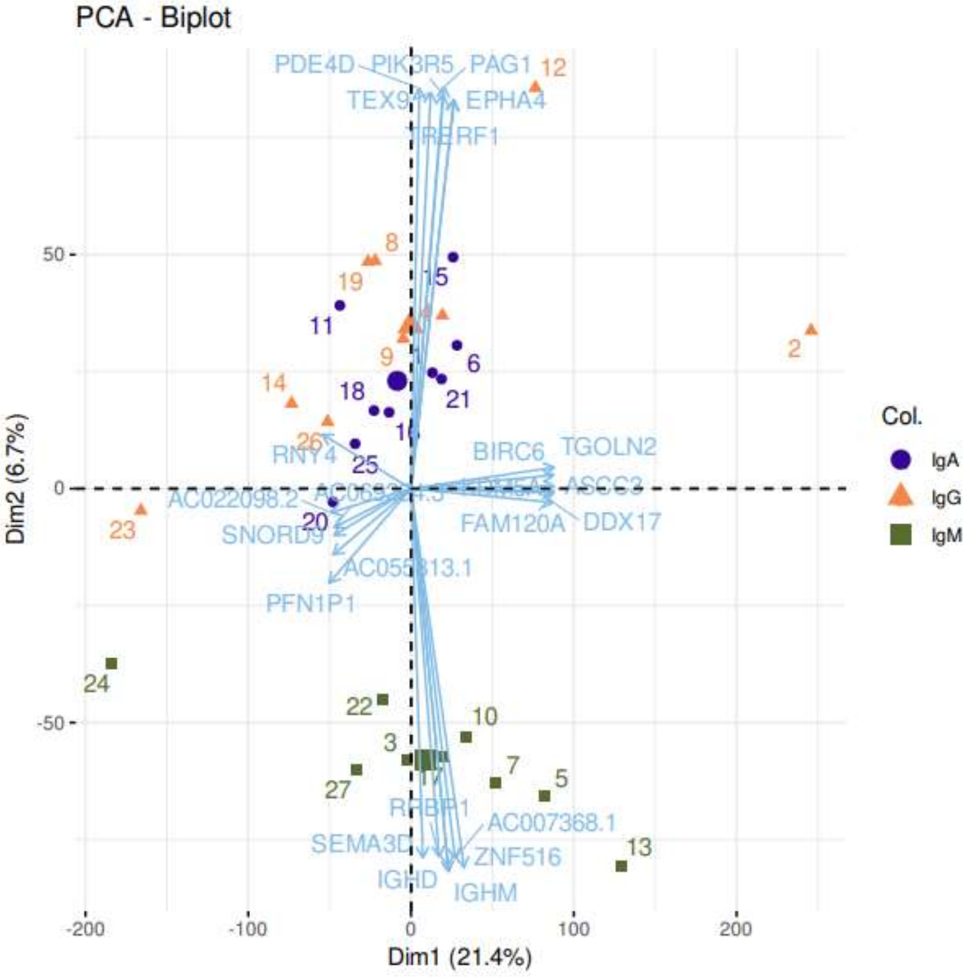

A: Panel 1

Target	Fluorochrome	Clone Name	Provider
**CD138**	**V450**	**MI15**	**BD**
**CD38**	**cFluor R720**	**HIT2**	**Cytek Biosciences**
CD199 (CCR9)	PE-Dazzle594	L053E8	Biolegend
CCR10	APC	6588-5	Biolegend
CD183 (CXCR3)	BV605	G025H7	Biolegend
**IgA**	**PerCP-Vio700**	**IS11-8^{E}10**	**Miltenyi**
**IgA2**	**PE-Vio770**	**IS11-21^{E}11**	**Miltenyi**
**IgG**	**BUV496**	**G18-145**	**BD (Optibuild)**
CD184 (CXCR4)	APC-Vio 770	REA649	Miltenyi
Integrin β7	BV650	FIB504	BD
CD125	BV786	A14	BD
CD29 (integrin β1)	PE	TS2/16	Biolegend
CD267 (TACI)	BV711	1A1-K21-M22	BD
CD269 (BCMA)	PE-Cy5	19F2	Biolegend
CD25	BV421	2A3	BD
CD268 (BAFFR)	BB700	11C1	BD
CD49d (integrin α4)	BUV805	9F10	Biolegend
**CD20**	**cFluor V547**	**2H7**	**Cytek Biosciences**
**CD19**	**cFluor B532**	**H1B19**	**Cytek Biosciences**
**CD3**	**cFluor V420**	**SK7**	**Cytek Biosciences**
**CD14**	**cFluor B548**	**63D3**	**Cytek Biosciences**
**CD21**	**BUV563**	**B-ly4**	**BD (Optibuild)**
**CD27**	**BUV395**	**L128**	**BD**

B: Panel 2

Target	Fluorochrome	Clone Name	Provider
CD307d (FcRL4)	Alexa 647	A1	BD
**CD138**	**V450**	**MI15**	**BD**
**CD38**	**cFluor R720**	**HIT2**	**Cytek Biosciences**
CD307e (FcRL5)	BUV737	509F6	BD
**IgA**	**PerCP-Vio700**	**IS11-8^{E}10**	**Miltenyi**
**IgA2**	**PE-Vio770**	**IS11-21^{E}11**	**Miltenyi**
**IgG**	**BUV496**	**G18-145**	**BD (Optibuild)**
CD274 (PD-L1)	BV711	29^{E}.2A3	Biolegend
GPR183 (EBI2)	PerCP-Cy5.5	SA313E4	Biolegend
CD11b (ITGAM)	BV650	ICRF44	ThermoFisher Scientific
CD71 (TfR)	PE	CY1G4	Biolegend
CD95 (FAS)	PE-CF594	DX2	BD
CD43	BUV615	1G10	BD
CD11c (ITGAX)	PE-Cy5	3.9	Biolegend
CD32 (FcγRII)	PE-Cy5.5	6C4	ThermoFisher Scientific
MHC Class II (HLA-DR)	BUV805	LN3	ThermoFisher Scientific
**CD20**	**cFluor V547**	**2H7**	**Cytek Biosciences**
**CD19**	**cFluor B532**	**H1B19**	**Cytek Biosciences**
**CD3**	**cFluor V420**	**SK7**	**Cytek Biosciences**
**CD14**	**cFluor B548**	**63D3**	**Cytek Biosciences**
**CD21**	**BUV563**	**B-ly4**	**BD (Optibuild)**
**CD27**	**BUV395**	**L128**	**BD**
**CD27**	**BUV395**	**L128**	**BD**
